# Aging with Purpose and Justice: What Recent Evidence from the Special Issue Tells Us and What It Leaves Unsaid

**DOI:** 10.3390/ejihpe15100205

**Published:** 2025-10-10

**Authors:** Paulo Santos-Costa, Liliana B. Sousa, Manuela Vilar

**Affiliations:** 1 The Health Sciences Research Unit: Nursing (UICISA: E), Nursing School of the University of Coimbra, 3004-011 Coimbra, Portugal; 2 Neuropsychological Assessment and Ageing Processes (NAAP) Group, Center for Research in Neuropsychology and Cognitive and Behavioral Intervention (CINEICC), Faculty of Psychology and Educational Sciences, University of Coimbra, 3000-115 Coimbra, Portugal

## Introduction

While population aging may be celebrated as a public health and social progress success story, it is a more nuanced experience than this, and aging without well-being is a hollow victory. As the United Nations’ 2030 Agenda for Sustainable Development states, “leaving no one behind” requires that older people are not simply included in health and social systems; it refers to the recognition of older people as rights bearing citizens who can contribute to society, make meaning, and grow.

Similarly, the Decade of Healthy Ageing (2021–2030) led by the World Health Organization (WHO, 2020) calls for courageous action in four areas: (i) transforming the way we think about age and aging; (ii) creating communities that support and optimize the unique contributions and capabilities of older persons; (iii) delivering integrated care; and (iv) ensuring access to long term care when needed. This Special Issue, called “Health and Well-Being among Older Adults: Current Trends and Future Challenges”, includes nine contributions from over a dozen European and Asian countries and provides practical accounts of both what has been achieved as well as questions still to be answered.

One clear contribution is the recognition that digital inclusion is now inseparable from social inclusion. Eto and Yamatsu in Japan showed that eHealth literacy strengthens older adults’ social activity, while Parra-Sánchez et al. in Spain revealed how geography and gender shape the perceived benefits of technology among elders living with visual impairment. Fernández-Piqueras et al., also in Spain, highlighted how uneven digital competence generates new vulnerabilities and proposed intergenerational learning as a pathway to inclusion. These studies speak directly to Sustainable Development Goal (SDG) 10 (Reduced Inequalities) by confronting a new form of inequity, the “digital divide”, and to the WHO Decade’s call to foster environments that support older people’s abilities (WHO, 2020). However, one key issue still unanswered is the role of the digital infrastructure, platform design, accessibility considerations, or affordability, in creating systematic exclusion. For there to be equitable aging, future research and policy must work to support older adults’ digital skills but also pay attention to the systemic biases embedded in the technologies they are asked to use.

A second thread concerns cognitive health and prevention, aligning closely with SDG 3 (Good Health and Well-Being). Bouhaben et al. in Spain provided evidence that olfactory identification may serve as a cost-effective biomarker for early cognitive decline, while Tsiakiri et al. in Greece demonstrated that computerized cognitive training can stabilize decline in older adults with minor neurocognitive disorders. Tomás et al., drawing on data from more than 61,000 older adults, confirmed that loneliness, pain, and frailty are decisive predictors of quality of life, with loneliness emerging as the most consistent determinant. These studies contribute to the Decade’s call for integrated, person-centered care (WHO, 2020) and align with the Global Coalition on Aging (GCOA, 2022) framework’s insistence that age-related conditions, whether frailty or cognitive decline, should not be passively accepted as inevitable, but proactively managed with preventive strategies. Yet, here again, questions remain. How can low-cost biomarkers like olfactory testing be embedded in public health systems that often underfund geriatric care? What forms of cognitive training are scalable and culturally adaptable beyond research contexts? And why do health systems continue to privilege frailty and pain over loneliness, despite robust evidence of its corrosive effects within this cohort?

The Special Issue also insists that well-being in later life extends beyond health to questions of sexuality, meaning, and spirituality. Von Humboldt et al. studying older adults in Portugal and Spain, showed that sexual well-being remains diverse and culturally shaped, challenging the persistent ageist assumption that sexuality fades into irrelevance in later life. Cojocaru et al. in Romania highlighted the sources of meaning for institutionalized elders, from spirituality to intergenerational value transmission, underscoring that existential and relational needs remain vital even in contexts of dependency. A study by Volkos et al. conducted in Greece further demonstrated the links between loneliness, health behaviors, and biological markers, reinforcing that emotional and physical dimensions of aging cannot be separated. These contributions directly advance SDG 5 (Gender Equality) by recognizing sexuality and intimacy as ongoing in later life, and they align with SDG 16 (Peace, Justice, and Strong Institutions) by exposing how institutional settings often neglect the psychosocial dimensions of aging. Yet, they raise uncomfortable questions. If sexuality and spirituality are empirically shown to matter, why are they absent from most health and social care models? Are they taught in-depth during formal training of healthcare professionals such as nurses and doctors? How long can policymakers and practitioners ignore these dimensions without reproducing ageism in subtler forms?

Geographically, the scope of this Special Issue is notable. It encompasses local studies from Japan, Greece, Spain, Portugal, and Romania, alongside cross-national analysis of 28 European countries and Israel conducted by Tomás et al. This diversity responds to SDG 17 (Partnerships for the Goals) by demonstrating how comparative and collaborative research can generate insights that no single country could produce alone. At the same time, it underscores how unevenly the research map is populated. We hear little about aging in Africa, Latin America, or the Global South, despite these regions experiencing some of the fastest demographic transitions. If the 2030 Agenda is truly global, then scholarship must urgently extend beyond high-income and European contexts to capture the plurality of aging experiences.

Across themes, the contributions collected here affirm two central points. First, that aging is irreducibly multidimensional, requiring integrated approaches across health, psychology, education, and social policy. Second, that progress is possible, but only if research findings are translated into bold policies that align with global agendas. Digital literacy must be embedded as a citizenship right, cognitive health interventions must be scaled and equitably funded, loneliness must be recognized as a health condition, and sexuality and spirituality must be legitimized in care frameworks.

At the same time, this Special Issue reveals blind spots; namely, the economic dimensions of aging, the intersections of ethnicity and migration, and the voices of the oldest-old remain underexplored. These omissions should not be seen as shortcomings but as an invitation for further scholarship that continues to push the boundaries of how we understand aging.

Finally, the contributions to this Special Issue are not only empirical studies but responses to the global call for action on aging. They align with specific Sustainable Development Goals, advance the priorities of the WHO Decade of Healthy Ageing (WHO, 2020), and reinforce the International Council on Active Aging (ICAA, 2016) and GCOA (2022) insistence that ageism, neglect, and inequity cannot be tolerated in aging societies.

They also leave us with critical challenges; namely, to broaden the geographies of research, to take seriously the non-biomedical dimensions of aging, and to build policies that match the complexity of older adults lived experiences. In highlighting both contributions and unanswered questions, this Special Issue demonstrates that healthy aging is not simply a technical or clinical challenge, but a profound societal project, and one that demands courage, imagination, and above all, justice.

## Abbreviations

The following abbreviations are used in this manuscript:

GCOA	Global Coalition on Aging
ICAA	International Council on Active Aging
SDG	Sustainable Development Goal
WHO	World Health Organization
